# Advisory Committee on Immunization Practices Recommended Immunization Schedule for Adults Aged 19 Years or Older — United States, 2014

**Published:** 2014-02-07

**Authors:** Carolyn B. Bridges, Tamera Coyne-Beasley, Advisory Committee on Immunization Practices (ACIP), ACIP Adult Immunization Work Group

**Affiliations:** 1 Immunization Services Division, National Center for Immunization and Respiratory Diseases, CDC; 2 Division of General Pediatrics and Adolescent Medicine, University of North Carolina, Chapel Hill

Vaccines are recommended for adults on the basis of their age, prior vaccinations, health conditions, lifestyle, occupation, and travel. Reasons for current low levels of vaccination coverage for adult vaccines are multifactorial and include limited awareness among the public about vaccines for adults and gaps in incorporation of regular assessments of vaccine needs and vaccination into routine medical care (1–4). Updated standards for immunization of adults were approved by the National Vaccine Advisory Committee (NVAC) in September 2013 (5). These standards acknowledge the current low levels of vaccination coverage among adults and the role that all health-care providers, including those who do not offer all recommended adult vaccines in their practices, have in ensuring that their patients are up-to-date on recommended vaccines. NVAC recommends that providers assess vaccination needs for their patients at each visit, recommend needed vaccines, and then, ideally, offer the vaccine or, if the provider does not stock the needed vaccines, refer the patient to a provider who does vaccinate. Vaccinating providers should also ensure that patients and their referring health-care providers have documentation of the vaccination.

*Recommendations for routine use of vaccines in children, adolescents, and adults are developed by the Advisory Committee on Immunization Practices (ACIP). ACIP is chartered as a federal advisory committee to provide expert external advice and guidance to the Director of the Centers for Disease Control and Prevention (CDC) on use of vaccines and related agents for the control of vaccine-preventable diseases in the civilian population of the United States. Recommendations for routine use of vaccines in children and adolescents are harmonized to the greatest extent possible with recommendations made by the American Academy of Pediatrics (AAP), the American Academy of Family Physicians (AAFP), and the American College of Obstetrics and Gynecology (ACOG). Recommendations for routine use of vaccines in adults are harmonized with recommendations of AAFP, ACOG, and the American College of Physicians (ACP). ACIP recommendations adopted by the CDC Director become agency guidelines on the date published in the* Morbidity and Mortality Weekly Report (MMWR). *Additional information regarding ACIP is available at http://www.cdc.gov/vaccines/acip*.

A recommendation by a patient’s health-care provider for needed vaccines is a strong predictor of patients receiving recommended vaccines (6,7). Other interventions to improve vaccination rates have been summarized in the *Community Guide* (http://www.thecommunityguide.org/vaccines/index.html) and include systems changes, such as routine screening and offering of vaccines and implementation of reminder/recall systems (8).

Because many adult patients might consult more than one health-care provider and also might be vaccinated at the workplace, pharmacy, or other location, documentation of vaccinations in immunization information systems (IIS) (i.e., vaccine registries) is important to ensure that a patient’s complete vaccination history is available to all of his/her providers. In addition, some vaccines require more than 1 dose with specified time intervals between doses (e.g., hepatitis B vaccine 3-dose series) or are recommended for certain adult populations only if adults were not vaccinated as children (e.g., measles-mumps-rubella [MMR] vaccine). IIS are managed by state or city immunization programs; contact information about these systems is available at http://www.cdc.gov/vaccines/programs/iis/contacts-registry-staff.html.

The Advisory Committee on Immunization Practices (ACIP) annually reviews and updates the *Recommended Immunization Schedule for Adults Aged 19 Years or Older*. This schedule provides a brief summary of ACIP recommendations for the use of vaccines routinely recommended for adults in the form of two figures, footnotes for each vaccine, and a table that includes primary contraindications and precautions.

In October 2013, ACIP approved the *Recommended Immunization Schedule for Adults Aged 19 Years or Older* for 2014. This schedule was also reviewed and approved by the American Academy of Family Physicians, the American College of Physicians, the American College of Obstetricians and Gynecologists, and the American College of Nurse-Midwives. The primary updates for the 2014 schedule include adding *Haemophilus influenzae* type b (Hib) vaccine to the figures and updating information in the footnote about persons for whom Hib vaccine is recommended; adding information to the influenza vaccine footnote and contraindications table regarding the newly licensed recombinant influenza vaccine (RIV) and information about the use of RIV and inactivated influenza vaccine (IIV) among persons with egg allergies; moving the footnote for pneumococcal conjugate vaccine (PCV13) recommendations before the pneumococcal polysaccharide vaccine (PPSV23) recommendations because PCV13 should be administered first among persons for whom both vaccines are recommended; and clarifying information about the timing of the second and third doses of human papillomavirus (HPV) vaccine, use of meningococcal vaccines among adults, and recommendations for tetanus, diphtheria, acellular pertussis (Tdap) and tetanus and diphtheria (Td) vaccines (9–10).

Because of space limitations, many details of the full ACIP recommendations for each vaccine are not included in the schedule, and interested health-care providers should refer to the full ACIP recommendations. In addition, changes in recommendations for specific vaccines might occur between annual updates to the adult immunization schedule. ACIP recommendations for specific vaccines are available at http://www.cdc.gov/vaccines/hcp/acip-recs/index.html. Information on reporting vaccine-related adverse events is available online at http://www.vaers.hhs.gov or by telephone at 800-822-7967.

The full 2014 schedule is published in the *Annals of Internal Medicine* (11). This year, the figures, footnotes, and tables are not being published in *MMWR*, but will be posted and maintained on the CDC website at http://www.cdc.gov/vaccines/schedules to facilitate updating the schedule during the year, if needed. If errors or omissions are detected after publication of the pediatric or adult immunization schedules, CDC posts revised versions. CDC encourages organizations that have previously relied on copying and posting portable document format (PDF) files of the schedules to their websites to instead use “content syndication” to ensure that current and accurate immunization schedule information appears on each organization’s website. This one-time step ensures that websites display current yearly schedules as soon as they are published or revised. Instructions for copying and placing syndication code are available at http://www.cdc.gov/vaccines/schedules/syndicate.html. CDC offers technical assistance for organizations implementing this form of content syndication. For assistance, readers can complete the e-mail form on the CDC’s National Center for Immunization and Respiratory Diseases (NCIRD) web support page (http://www.cdc.gov/vaccines/about/contact/web_problem_form.htm), and an NCIRD web team member will contact them to provide assistance.

## Changes for 2014

### Footnotes

- Hib vaccine recommendations were updated. The vaccine is recommended for certain adults at increased risk for Hib who have not received the Hib vaccine before. Adults who have had a successful hematopoietic stem cell transplant are recommended to receive a 3-dose series of Hib vaccine 6–12 months after transplant regardless of prior Hib vaccination. Prior Hib vaccine guidance recommended that Hib vaccination of persons infected with human immunodeficiency (HIV) be considered, but updated guidance no longer recommends Hib vaccination of previously unvaccinated adults with HIV infection because their risk for Hib infection is low.
- Information on RIV and the use of RIV and IIV among egg-allergic patients was added to the footnote and indicates that RIV or IIV can be used among persons with hives-only allergy to eggs. RIV contains no egg protein and can be used among persons aged 18 through 49 years who have egg allergy of any severity.
- The Td/Tdap vaccine footnote was edited to harmonize language used in the pediatric immunization schedule. A single dose of Tdap vaccine is recommended for previously unvaccinated persons aged 11 years or older, and a Td booster should be administered every 10 years thereafter. Pregnant women continue to be recommended to receive 1 dose of Tdap vaccine during each pregnancy, preferably during 27–36 weeks’ gestation, regardless of the interval since prior dose of Tdap or Td vaccine.
- Information was added to the HPV footnote to clarify the timing between the second and third doses and to harmonize language between the pediatric and adult immunization schedules; no changes in recommendations were made.
- The HPV vaccine and the zoster vaccine footnotes were simplified, with removal of the bullet regarding health-care personnel (HCP). Being a health-care worker is not a specific indication for these vaccines, but they should be given to HCP and others who meet age and other indications for these vaccines. Information on HCP vaccination for all vaccines is available at http://www.cdc.gov/mmwr/preview/mmwrhtml/rr6007a1.htm.
- Because PCV13 is recommended to be administered before PPSV23 among persons for whom both vaccines are recommended, the PCV13 footnote now precedes the PPSV23 footnote and includes wording to remind providers of the appropriate order of these vaccines when both are indicated.
- The meningococcal vaccine footnote was edited to clarify which persons need either 1 or 2 doses of vaccine and to provide greater clarity regarding which patients should receive the meningococcal conjugate versus the meningococcal polysaccharide quadrivalent vaccines.
- No changes or minor clarifications were made to the MMR, hepatitis A, or hepatitis B vaccine footnotes; no changes in recommendations were made.

### Figures

- For Figures 1 and 2, a row for Hib vaccine was added, and the PCV13 vaccine row was moved before PPSV23 as a reminder that PCV13 vaccines should be administered first among patients for whom both vaccines are recommended.

## Contraindications and precautions table

- The contraindications and precautions table was updated to include information on RIV, an influenza vaccine that contains no egg protein and is indicated for persons aged 18 through 49 years.
- The Hib vaccine was added to the table.
